# Case Report: Endovascular therapy for an iatrogenic vertebrojugular arteriovenous fistula and pseudoaneurysm induced by multiple vascular procedures

**DOI:** 10.3389/fcvm.2025.1493342

**Published:** 2025-04-04

**Authors:** Huai-Xue Mi, Chun-Mei Guo, Shan-Liang Chen, Jun Zhang, Zhi Gao, Li Hongxin, Ju Han

**Affiliations:** ^1^ Department of Cardiovascular Surgery, The First Affiliated Hospital of Shandong First Medical University & Shandong Provincial Qianfoshan Hospital, Jinan, China; ^2^ Department of Cardiovascular Surgery, Shandong Provincial Hospital Affiliated to Shandong First Medical University, Jinan, China; ^3^ Department of Neurology, The First Affiliated Hospital of Shandong First Medical University & Shandong Provincial Qianfoshan Hospital, Jinan, China

**Keywords:** vertebrojugular arteriovenous fistula, vertebrojugular arteriovenous pseudoaneurysm, endovascular therapy, iatrogenic injury, vascular procedures

## Abstract

Vertebrojugular arteriovenous fistula (VJAVF) and vertebral artery pseudoaneurysm (VAPA) are usually caused by iatrogenic and penetrating traumas. The concurrence of these two entities originating from different ostia of the vertebral artery (VA) is extremely rare. The history of repeated open-heart surgery and the application of central venous catheterization during anesthesia increased the risk of VA injuries and its complications of the VJAVF and VAPA. The patient, who complained of dizziness, was initially diagnosed with bradycardia and aortic paravalvular leak. However, the symptoms persisted even after permanent pacemaker implantation and transcatheter closure of the aortic paravalvular leak. Ultrasonography, CT angiography, and intraoperative right subclavian arteriography showed a VJAVF originating from the right VA and draining into the right internal jugular vein. A VAPA originated from another ostium of the right VA without a drainage vessel. Using the endovascular technique, a 4 mm stent graft was deployed in a 3.6 mm VA to cover both the VJAVF and the VAPA ostia. The symptoms of dizziness disappeared. The VJAVF and VAPA were completely sealed without recurrence at the 6-month follow-up.

## Introduction

Penetrating vertebral artery (VA) injuries (VAIs) are rare. Vertebrojugular arteriovenous fistula (VJAVF) and vertebral artery pseudoaneurysm (VAPA) are late complications of VAIs caused by iatrogenic and penetrating traumas. Because of their rarity, complex anatomy, and difficult surgical exposures, endovascular treatment has emerged as a preferable alternative to surgical treatment (1). Dizziness is a common symptom with many possible causes. VJAVF and VAPA should be considered in patients with dizziness who received multiple vascular procedures, such as central venous catheterization and diagnostic cerebral angiography.

## Case presentation

A 55-year-old female patient complaining of dizziness and chest tightness was admitted to our hospital. The patient had a history of aortic and mitral valve replacement 14 years before and redo aortic valve replacement 3 years before due to mechanical valve restenosis. In the previous open-heart surgeries, right internal jugular vein (IJV) catheterization was routinely performed for the anesthesia and perioperative management. During anesthesia, multiple attempts at IJV catheterization were made using the anatomic landmark technique without the guidance of ultrasound; however, the number of attempts was not known.

The symptoms of dizziness started after the redo surgery. It was initially thought to be the result of bradycardia with a minimum heart rate of 30 beats/min. However, the symptoms persisted after the placement of a permanent pacemaker. Then, the symptoms were attributed to an aortic paravalvular leak, and a transcatheter closure of the leak was subsequently performed; however, still the symptoms persisted.

**Figure 1:**
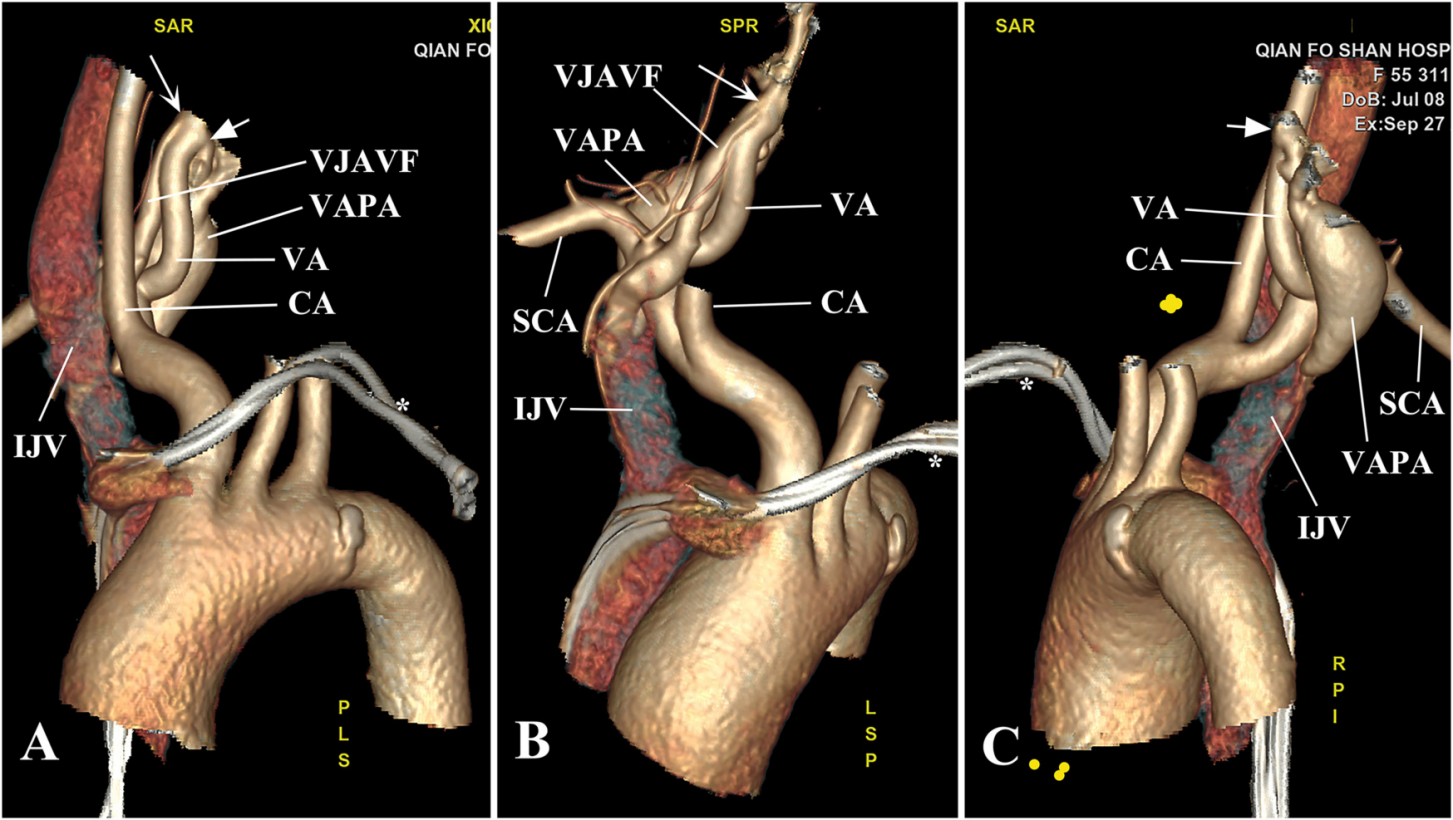
Anatomic location of the VJAVF and VAPA. **(A)** CTA revealed a VJAVF originating from the V1 segment of the right VA (arrow) that went down deeply behind the right CA and drained into the right IJV from behind. **(B)** The long course of the VJAVF is shown. **(C)** A banana-like VAPA, measuring 38 mm × 17 mm in size, originated from the opposite wall of the right VA (arrowhead) and extended down to the level below the right SCA without a drainage vessel. *indicates pacemaker wires. CA, carotid artery; IJV, internal jugular vein; SCA, subclavian artery; VA, vertebral artery; VAPA, vertebral artery pseudoaneurysm; VJAVF, vertebrojugular arteriovenous fistula.

A significant carotid artery bruit was auscultated incidentally on the right side of the neck. After evaluation with color Doppler ultrasonography and computed tomographic angiography (CTA), this was determined to be VJAVF and VAPA (Figure 1). The most common causes of dizziness include vertebrobasilar cerebrovascular disease, migraine-associated vertigo, multiple sclerosis, and space-occupying lesions (2). VJAVF may result in vertebrobasilar ischemia and dizziness due to the arterial steal phenomenon.

**Figure 2:**
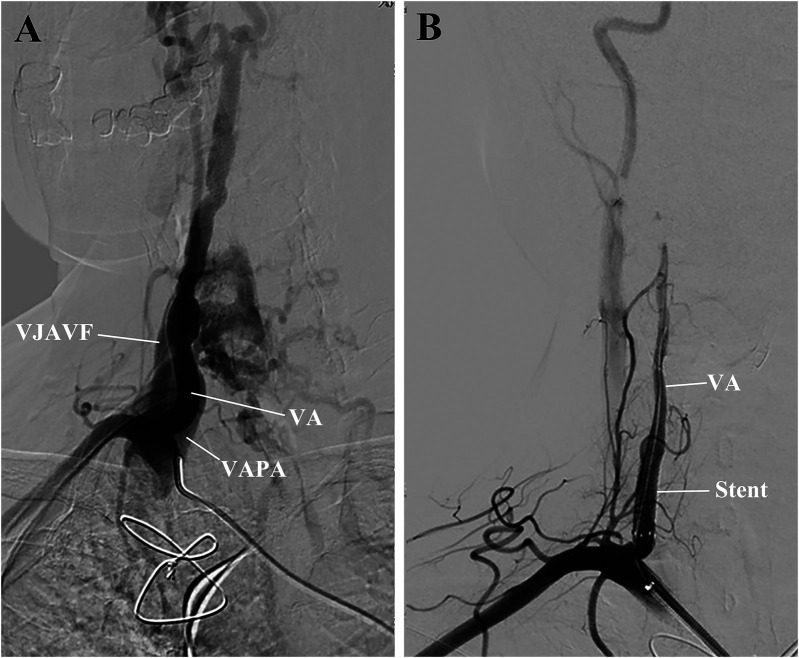
Preoperative and postoperative right subclavian arteriography. **(A)** The exact origin of the VJAVF and VAPA and the diameter of the VA were carefully determined. Collateral circulation was noted in this area. **(B)** After the procedure, the angiograms revealed a complete sealing of the VJAVF and VAPA, improved distal flow of the VA, and reduced collateral circulation. VA, vertebral artery; VAPA, vertebral artery pseudoaneurysm; VJAVF, vertebrojugular arteriovenous fistula.

After informed consent was obtained from the patient, a 6 Fr Neuron Max catheter was introduced into the right subclavian artery via the left femoral approach, and a selective right subclavian arteriography was performed (Figure 2A). With the aid of a 6 Fr guiding catheter and soft guidewire, the Neuron Max catheter was advanced to the V1 segment of the right VA, while the soft wire was advanced into the distal V2 segment**.** A VIABAHN delivery catheter loaded with an endovascular prosthesis (W.L. Gore & Associates, Inc., USA) was advanced into the distal V2 segment over the wire. A 4 mm stent graft coated with a heparin bioactive surface was deployed in a 3.6 mm VA to cover both the VJAVF and the VAPA ostia (Figure 2B).

**Figure 3:**
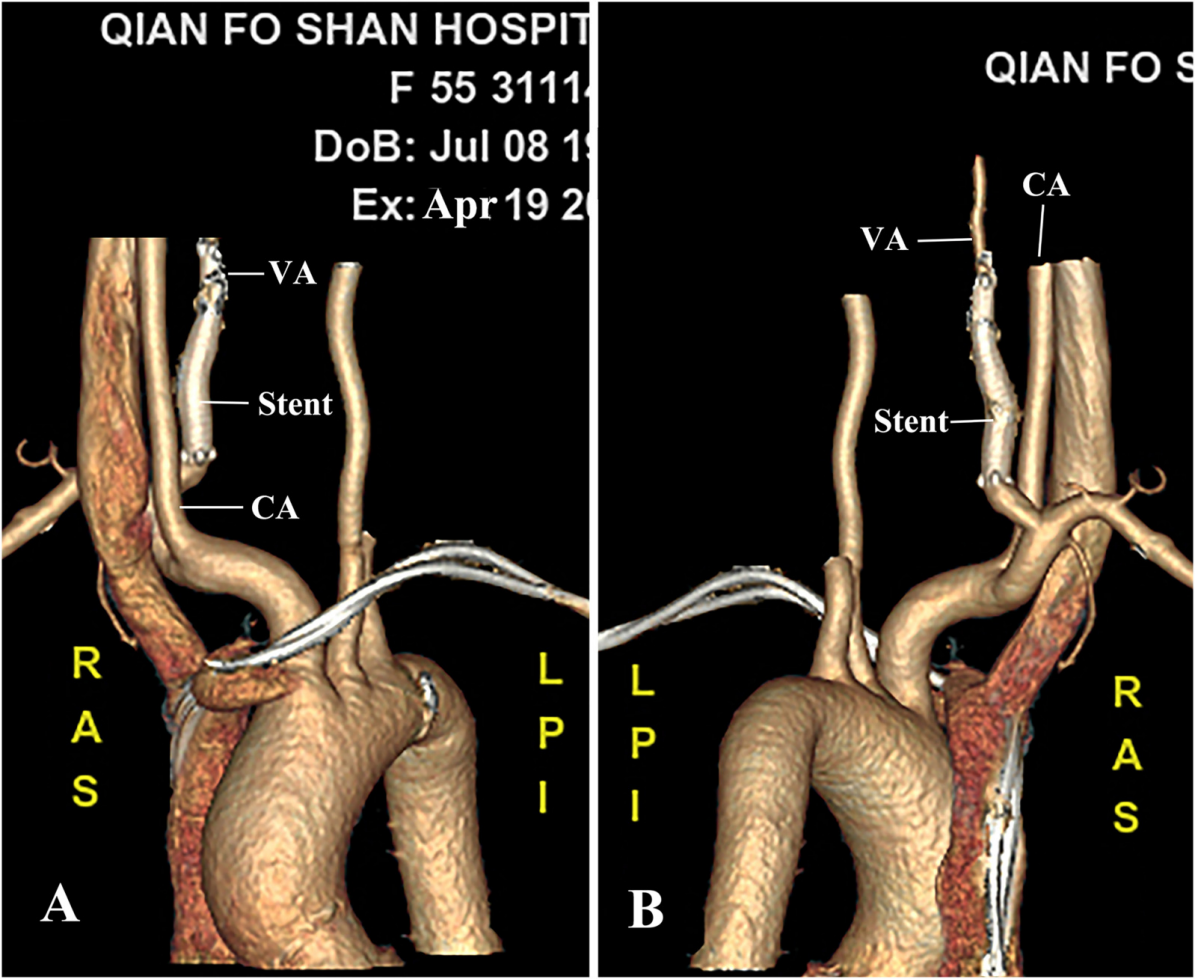
CTA at 6-month follow-up. **(A,B)** Better luminal filling of the VA was observed on CTA without intra-stent stenosis and recurrence of the fistula or pseudoaneurysm. CA, carotid artery; VA, vertebral artery.

The patient woke up without any neurologic deficits. She was discharged home 7 days postoperatively with a prescription for 100 mg aspirin and 75 mg clopidogrel per day. At the 6-month follow-up, the VA maintained patency without recurrence of VJAVF or VAPA (Figures 3A,B).

## Discussion

VAI is a rare problem that is typically associated with cervical penetration, blunt trauma, or iatrogenic reasons. The incidence of VAIs in the civilian and military populations was 3.1% and 0.3%, respectively (1). There are far fewer cases of VAIs accompanied by VJAVF or VAPA formation.

The most common complication associated with IJV catheterization was found to be a puncture of the carotid artery, with an incidence in the range of 2%–9.9%. Some of the punctures form a carotid-jugular arteriovenous fistula. Although VJAVF or VAPA are rarer than carotid-jugular arteriovenous fistula, multiple vascular procedures, such as repeated IJV catheterization, may increase the risks of VJAVF and VAPA. In patients with difficult IJV catheterization, ultrasound guidance for IJV cannulation may help minimize the number of attempts required and the risk of complications.

VAIs most commonly involve the V2 segment of the VA but rarely involve the V1 segment (1, 3). It is not uncommon for these two lesions to coexist. The concurrence of these two entities originating from different ostia of the V1 segment is extremely rare. Disorders may result in vertebrobasilar ischemia, dizziness, diplopia, and cephalgia due to arterial steal (1, 3). Stenting or repair of VJAVF or VAPA have been less frequently performed and reported (4.7%) (1, 4), let alone simultaneous stenting and sealing of both lesions.

In this case, the worsening symptoms of dizziness had been initially suspected to be due to redo aortic valve replacement, bradycardia, or aortic paravalvular leak. For differential diagnosis, dizziness occurred not only in patients with cardiovascular and cerebrovascular diseases but also in patients with internal jugular catheterization, which can cause VJAVF and VAPA. These two lesions, with different ostia of the VA, may be caused by different VAIs resulting from multiple manipulations of the central venous catheterization during anesthesia.

Treatment of VJAVF or VAPA includes surgical treatment, embolization with detachable balloons, coils or other agents, and stents (1). Conventionally, these entities have mostly been treated by surgery (4). The deep position and complex anatomy of VJAVF and VAPA make these complications hard to treat. Surgery generally requires a large and complex exposure, which is quite invasive (3, 5).

Endovascular interventions are considered more secure, less painful, and less invasive (3). Both surgical and transcatheter embolization often require sacrificing the VA, which can result in ischemic damage to the brain. Reconstruction of the VA with a stent is mandatory. In this case, the main advantage of the treatment is to seal both the VJAVF and VAPA while maintaining VA patency.

To minimize the occurrence of VJAVF or VAPA, anesthetists should take note of the following points: (1) it is crucial to avoid a too-deep or lateral insertion of the puncture needle during internal jugular catheterization; (2) the use of ultrasound has been advocated to locate the vein to prevent inadvertent VAI and its late complications of VJAVF and pseudoaneurysm; and (3) VJAVF and VAPA can be caused iatrogenically by multiple vascular procedures.

## Data Availability

The original contributions presented in the study are included in the article/Supplementary Material, further inquiries can be directed to the corresponding authors.
