# Erratum: Case Report: Endovascular therapy for an iatrogenic vertebrojugular arteriovenous fistula and pseudoaneurysm induced by multiple vascular procedures

**DOI:** 10.3389/fcvm.2025.1628458

**Published:** 2025-05-27

**Authors:** Frontiers Production Office

**Affiliations:** Frontiers Media SA, Lausanne, Switzerland

**Keywords:** vertebrojugular arteriovenous fistula, vertebrojugular arteriovenous pseudoaneurysm, endovascular therapy, iatrogenic injury, vascular procedures

An Erratum on Case Report: Endovascular therapy for an iatrogenic vertebrojugular arteriovenous fistula and pseudoaneurysm induced by multiple vascular procedures By Mi H-X, Guo C-M, Chen S-L, Zhang J, Gao Z, Hongxin L, Han J (2025). Front. Cardiovasc. Med. 12:1493342. doi: 10.3389/fcvm.2025.1493342

Due to a production error, the article acceptance date should be 20th March 2025 instead of 10th December 2024, and the volume number should be 12 instead of 3.

The publisher apologizes for this mistake. The original version of this article has been updated.

